# Assessment of genomic and antifungal properties of *Lactococcus garvieae* ZB15 isolated from Zhenba bacon

**DOI:** 10.3389/fmicb.2025.1610971

**Published:** 2025-06-26

**Authors:** Guojin Li, Xingyu He, Jiao Li, Yuwen Jian, Lianxu Zhu, Shanshan Wang, Wenxian Zeng, Tao Zhang, Hongzhao Lu, Ling Wang

**Affiliations:** ^1^School of Biological Science and Engineering, Shaanxi University of Technology, Hanzhong, China; ^2^Hanzhong Agricultural Technology Extension and Training Center, Hanzhong, China; ^3^Shaanxi University Engineering Research Center of Quality Improvement and Safety Control of Qinba Special Meat Products, Hanzhong, China; ^4^Shaanxi Union Research Center of University and Enterprise for Zhenba Bacon, Hanzhong, China; ^5^Qinba State Key Laboratory of Biological Resources and Ecological Environment, Hanzhong, Shaanxi, China

**Keywords:** *Lactococcus garvieae*, genome, probiotic, antifungal effect, lactic

## Abstract

**Introduction:**

Lactic acid bacteria (LAB) have attracted significant interest as natural antimicrobials for food safety and preservation. Some LAB strains effectively inhibit food-borne molds and yeasts responsible for spoilage and mycotoxin contamination. However, the antifungal effect of *Lactococcus garvieae* remains largely unexplored.

**Methods:**

This study investigates the properties of *L. garvieae* ZB15 isolated from Zhenba bacon, emphasizing its genetic safety and antimicrobial potential. The genomic information and antifungal properties of Lactococcus garvieae strain ZB15 were comprehensively analyzed using whole-genome sequencing and in vitro experiments.

**Results:**

Genomic analysis confirmed the absence of virulence and antibiotic resistance genes, along with minimal prophage presence, underscoring its safety for application. The strain exhibited strong self-aggregation, co-aggregation, and hydrophobic properties, suggesting its potential for intestinal adhesion. The cell-free supernatant (CFS) of *L. garvieae* ZB15 demonstrated notable antifungal activity, achieving over 50% inhibition against four fungal strains at 60 mg/mL. The antifungal compounds in the CFS retained high activity after thermal treatment at 100°C for 1 hour, highlighting their thermal stability. Additionally, enzymatic treatments with trypsin, pepsin, and proteinase K, along with neutralization of organic acids, significantly reduced the antifungal activity, indicating the involvement of proteinaceous compounds and organic acids. Catalase treatment partially affected antifungal activity, suggesting hydrogen peroxide as a contributing factor.

**Discussion:**

*Lactococcus garvieae* ZB15 possessed probiotic characteristics, including strong adhesion properties and pathogen exclusion capabilities. The strain’s genetic safety, lack of antibiotic resistance genes, and ability to produce heat-stable antifungal compounds highlighted its potential as a natural food preservative.

## 1 Introduction

Lactic acid bacteria (LAB) represent a diverse group of Gram-positive, non-spore-forming, facultative anaerobes that predominantly produce lactic acid through carbohydrate fermentation (Navarré et al., 2024). Ubiquitously distributed, LAB can be isolated from a wide variety of sources, including fermented foods such as pickles (Seddik et al., 2017), dairy products (Taye et al., 2021; Yu et al., 2011), vegetables (Bringel et al., 2005; Kim et al., 2008), meat products (Li et al., 2024), silage (Ellis et al., 2016), and wine (Ambe et al., 2024). Moreover, LAB form a critical component of the human and animal gastrointestinal and vaginal microbiota (Liu et al., 2021; Zhang et al., 2024; Zhong et al., 2022). The metabolic products of LAB have extensive applications in industries ranging from animal feed and aquaculture to food processing and storage, where their preservative effects and contributions to product stability and flavor are highly valued. To date, LAB comprises over 18 genera and more than 200 species, with prominent members including *Lactobacillus*, *Enterococcus*, *Lactococcus*, and *Leuconostoc* (Goldstein et al., 2015).

*Lactococcus garvieae* is a facultative anaerobic, Gram-positive bacterium found in diverse environments (Jantrakajorn et al., 2024). Initially identified as a fish pathogen, certain strains exhibit probiotic properties. For example, *L. garvieae* B301 reduces diarrhea and mortality in poultry while enhancing weight gain, suggesting its potential as a feed additive (Gradoville et al., 2018). *L. garvieae* DCC43, isolated from wild duck intestines, produces a circular bacteriocin with antibacterial properties (Kawada et al., 2016). Another strain from Mongolian sheep intestines improves gut barrier integrity and mitigates gut dysbiosis in mice infected with *Clostridium perfringens* (Wang et al., 2024). In poultry farming, *L. garvieae* supplementation enhances immune function, promotes growth, increases secretory IgA (SIgA) levels, and supports gut microbial balance by reducing *Escherichia coli* populations while increasing LAB abundance (Zhang et al., 2016).

Lactic acid bacteria exhibit antifungal activity through competitive exclusion, organic acid production, and secretion of antifungal peptides and metabolites such as hydrogen peroxide and bacteriocins (Rabaoui et al., 2023). Some LAB strains effectively inhibit food-borne molds and yeast responsible for spoilage and mycotoxin contamination. The introduction of *Lactobacillus kefiri* M4 into apple juice suppresses fungal growth *via* nutrient competition with fungal species (Sadiq et al., 2019). Fatty acids and acetate produced by *Lactiplantibacillus*, *Furfurilactobacillus milii*, and *Lentilactobacillus parabuchneri* inhibit the growth of *Candida sake*, *Saccharomyces bayanus*, and *Torulaspora delbrueckii* in milk by suppressing fungal hyphal development (Nasrollahzadeh et al., 2022). However, the antifungal effect of *L. garvieae* remain largely unexplored.

Zhenba bacon, a traditional fermented meat product from Shaanxi Province, China, is known for its distinct color and flavor, achieved through salting and smoking processes. These processes foster complex microbial communities typical of Chinese cured meats (Rubio et al., 2014). Within the microbiota of Zhenba bacon, we identified the *L. garvieae* ZB15 strain, which exhibits a high growth rate, robust acid production, and antifungal potential. However, its genomic properties and antifungal activity remain underexplored.

This study aims to characterize the genome of *L. garvieae* ZB15 and assess its antifungal properties through *in vitro* experiments. The findings will enhance understanding of its probiotic properties, contributing to the development of sustainable, safe, and effective food preservation strategies.

## 2 Materials and methods

### 2.1 Bacterial strain and growth conditions

*Lactococcus garvieae* ZB15 was isolated from Zhenba bacon during the curing process with characteristics of high growth rate and robust acid production (data not shown). The strain was preserved as a glycerol stock at −80°C, streaked onto MRS agar plates and incubated overnight at 37°C. A single colony was picked and incubated overnight at 37°C in 2 mL sterile MRS broth until the culture reached an OD600 of 2.0, after which it was stored at 4°C for subsequent use.

### 2.2 Genomic DNA extraction, whole genome sequencing, and assembly

Genomic DNA was extracted from *L. garvieae* ZB15 using the HiPure Bacterial DNA Kit (Magen, Guangzhou, China) following the manufacturer’s protocol (Khan et al., 2016). The extracted DNA was quantified using a NanoDrop spectrophotometer and subjected to quality control by gel electrophoresis. DNA libraries were prepared and sequenced using an Illumina/ONT platform at Genedenovo Biotechnology Co., Ltd (Guangzhou, China). Raw sequencing reads were assembled *de novo* using Falcon (v0.3.0) and further refined with Flye (v2.8.1-b1676). Gene annotation was conducted using multiple databases, including NCBI-Nr, UniProt/Swiss-Prot, Kyoto Encyclopedia of Genes and Genomes (KEGG), Gene Ontology (GO), and Clusters of Orthologous Groups (COG). Non-coding RNA elements were identified using rRNAmmer (v1.2), tRNAscan-SE (v1.3.1), and cmscan (v1.1.2). Gene islands and prophages were predicted using Island Path-DIMOB (v1.0.0) and PHAST (v2.0), respectively.

### 2.3 Carbohydrate fermentation

Carbohydrate metabolism of *L. garvieae* ZB15 was assessed using the API 50 CHL test kit (bioMérieux, Marcy l’Etoile, France) as previously described (Yetiman et al., 2024). The bacterial suspension was prepared in API 50 CHL medium and adjusted to the turbidity standard recommended by the manufacturer. The inoculated test strip was incubated at 37°C under anaerobic conditions, and metabolic activity was determined by color change due to pH shift. No treated medium was taken as the negative control.

### 2.4 Antibiotic susceptibility testing

The Kirby–Bauer disk diffusion method was used to assess the antibiotic susceptibility of *L. garvieae* ZB15 as previously described (Khan et al., 2019; Sirichoat et al., 2020). Briefly, a standardized bacterial suspension was spread onto Mueller-Hinton agar, and 15 antibiotic-impregnated disks (Hangzhou Microbial Reagent Company, Hangzhou, China) were placed on the surface. After incubation at 37°C for 24 h, inhibition zone diameters were measured and interpreted according to Clinical and Laboratory Standards Institute (CLSI) guidelines (Hindler and Stelling, 2007).

### 2.5 Auto-aggregation, co-aggregation and hydrophobicity

#### 2.5.1 Auto-aggregation assay

Auto-aggregation ability was assessed as described by Vasiee et al. (2019) and El-Deeb et al. (2020). *L. garvieae* ZB15 was cultured in MRS broth until reaching an OD_600_ of 2.0. The culture was centrifuged at 4,000 × *g* for 10 min. After discarding the supernatant, the cell pellets were washed three times with sterile phosphate-buffered saline (PBS; pH 7.2) and resuspended in PBS to an initial OD_600_ of 1.0 (A_0_). The bacterial suspension was incubated statically at 37°C for 5 h. The upper phase was gently transferred into a 96-well plate (100 ul per well) for the measurement of OD_600_ (A_1_). Auto-aggregation percentage was calculated as:


$$\mathrm{Auto}-\mathrm{aggregation}\mathrm{ability}\left(\%\right)=\left(1-\frac{\mathrm{A1}}{\mathrm{A0}}\right)\times 100\%$$

#### 2.5.2 Co-aggregation assay

Co-aggregation (CA) assay was assessed as previously described (Mohanty et al., 2019). Briefly, *L. garvieae* ZB15 culture was prepared in MRS broth, and *Escherichia coli* ATCC 25922 (*E. coli* ATCC 25922), *Escherichia coli* K88 (*E. coli K88*), *Staphylococcus aureus* CMCC 26002 (*S. aureus* CMCC 26002), and *Listeria monocytogenes* CICC 21635 (*L. monocytogenes* CICC 21635) were cultured in LB medium. Upon reaching an OD_600_ of 2.0, bacterial cells were harvested by centrifuge (4,000 *g*, 10 min). The cell pellets were washed three times and resuspended in PBS. Pathogen suspensions were adjusted to an OD600 of 0.4 (A_2_), while the *L. garvieae* ZB15 suspension was adjusted to an OD600 of 0.6 (A_3_). Equal volumes of the pathogen and *L. garvieae* ZB15 suspensions were mixed at a 1:1 (v/v) ratio in 1 mL sterile tubes and incubated statically at 37°C for 5 h. The upper phase was gently transferred into a 96-well plate (100 μL per well) for the measurement of OD_600_ (A_4_), and CA percentage was calculated as:


$$\mathrm{Co}-\mathrm{aggregation}\mathrm{ability}\left(\%\right)=\left(1-\frac{\mathrm{A4}}{\frac{\mathrm{A2}+\mathrm{A3}}{2}}\right)\times 100\%$$

#### 2.5.3 Hydrophobicity ability detection

Hydrophobicity was measured based on bacterial affinity to chloroform, following Steinberg et al. (2022) with modifications. The bacterial cells were then harvested by centrifugation, washed with sterile PBS, and resuspended in PBS to an OD600 of 0.4 (A_5_). Subsequently, 3 mL of the cell suspension was mixed with 1 mL of chloroform, vortexed vigorously for 30 s, and allowed to stand undisturbed to allow phase separation. After separation, 1 mL of the upper aqueous phase was carefully removed and transferred into a 96-well plate (100 μL per well), and the optical density of the remaining aqueous phase was measured at 600 nm (A_6_). Hydrophobicity was calculated as:


$$\mathrm{Hydrophobicity}\left(\%\right)=\left(\frac{A_{5}-A_{6}}{A_{5}}\right)\times 100\%$$

### 2.6 Characterization of fungal inhibition

#### 2.6.1 Fungal growth inhibition assay

Referring to the method Ali and Reddy described (Ali and Reddy, 2000), the antifungal activity of the cell-free supernatant (CFS) from *L. garvieae* ZB15 was evaluated against *Penicillium grisofulvum* Dierckx (*P. grisofulvum* Dierckx), *Talaromyces verruculosus* (*T. verruculosus*), *Fusarium verticillioides* (*F. verticillioides*), and *Aspergillus ochraceus* (*A. ochraceus*) (BeNa Culture Collection, Xinyang, Henan, China). Fungal spores were harvested and adjusted to an OD600 of 1.0 with PBS. Ten microliter fungal spores were inoculated into a well of 96-well plates containing 130 uL PDA medium with 60 ul serial dilutions of CFS (20–100 mg/mL). Plates were incubated at 28°C for 48 h, and OD_600_ was measured (A_E_). In the control group, an equivalent volume (60 μL) of PDA medium was supplemented to take the place of CFS for measurement of OD_600_ (Ac). For fungal inhibition rate (%) determination and calculated as:


$$\mathrm{Fungal}\mathrm{inhibition}\mathrm{rate}\left(\%\right)=\left(1-\frac{A_{\text{E}}}{A_{\text{C}}}\right)\times 100\%$$

#### 2.6.2 Thermal stability of CFS

Cell-free supernatant (60 mg/mL) was subjected to heat treatment at 60°C, 80°C, and 100°C for 1 h. Subsequently, heat-treated CFS were used for fungal growth inhibition assay, and untreated CFS served as the control.

#### 2.6.3 pH neutralization assay

To evaluate the role of organic acids, the pH of CFS (initial pH 4.5) was adjusted to pH 5.8 using 5 mol/L NaOH. Then, fungal growth inhibition assay was performed, and untreated CFS (pH 4.5) served as the control.

#### 2.6.4 Protease sensitivity assay

To assess proteinaceous antifungal compounds, CFS was treated with protease (Trypases, Pepsin and Protease K, 2 mg/mL) for 2 h, followed by enzyme inactivation at 100°C for 5 min. Then, fungal growth inhibition assay was performed, and untreated CFS taken as the control.

#### 2.6.5 Hydrogen peroxide sensitivity assay

To determine the role of hydrogen peroxide, CFS was treated with 10 mg/mL catalase for 2 h, followed by enzyme inactivation at 100°C for 5 min. Then, fungal growth inhibition assay was performed, and untreated CFS served as the control.

#### 2.6.6 Scanning electron microscopy (SEM) of fungal morphology

Fungal morphology following CFS treatment was examined using SEM (Hitachi Reguius 8100, Hangzhou Feyman Biotechnology Co., Hangzhou, China). Samples treated with 60 mg/mL CFS were prepared for imaging, and untreated CFS served as the control.

#### 2.6.7 Data analysis

All experiments were conducted independently in triplicate. Data were presented as mean values ± standard deviation (SD). Statistical analyses, including significance testing and Pearson correlation coefficients, were performed using SPSS 23 (IBM, Armonk, NY, USA). A *p*-value of less than 0.05 was considered statistically significant.

## 3 Result

### 3.1 Whole-genome sequencing and functional genomic characterization

The genomic features of *L. garvieae* ZB15 are presented in Table 1. Its genome consists of a single circular chromosome with a total length of 1,996,386 base pairs and a GC content of 38.94%. In addition, it contains genetic elements such as CRISPR arrays, prophages, repeat sequences, and gene islands. These genetic factors may contribute to the genomic plasticity of the strain and play a crucial role in its probiotic and antifungal potential.

**TABLE 1:** General genomic information of the strain *L. garvieae* ZB15.

Features	Values	Features	Values
Genome size	1,996,386 bp	Prophage	2
GC Content	38.94%	CRISPR array region	6
Number of protein coding sequences	1,902	Repeated sequence	72
Number of tRNAs	62	Tandem repeat	40
Number of rRNAs	16	Gene islands	6
Number of non-coding RNA	78		

**FIGURE 1:**
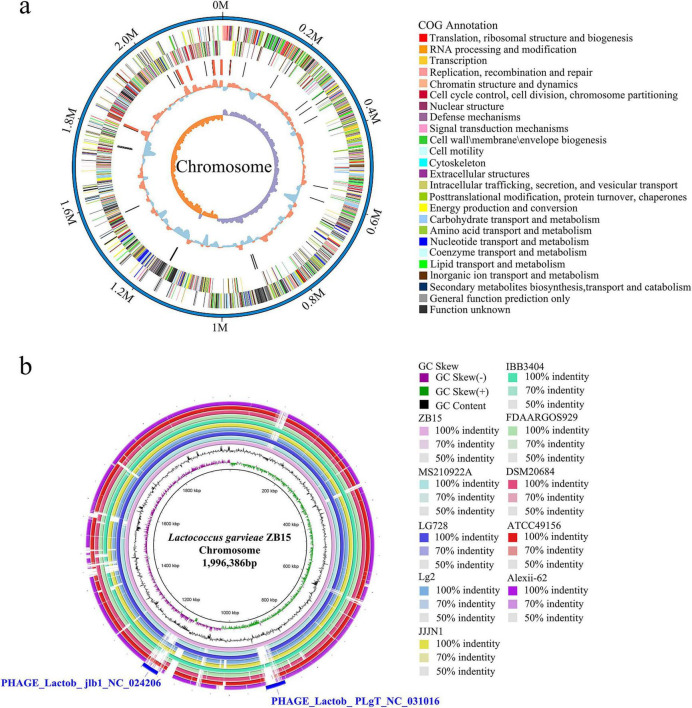
Circular genome map of *L. garvieae* ZB15. **(a)** The complete circular genome map of *L. garvieae* ZB15. **(b)** *L. garvieae* ZB15 genome BLAST comparison ring map.

The complete circular genome map of *L. garvieae* ZB15 was illustrated in Figure 1a. The outermost ring represents the genome size, with each scale mark denoting 0.2 Mb. The second and third rings displayed protein-coding sequences (CDS) on the positive and negative strands, respectively, with color-coded classifications based on COG. The fourth ring highlighted the locations of rRNA and tRNA genes, while the fifth ring showed GC content variations. Regions with GC content exceeding the genome-wide average were marked in red, with peak heights corresponding to the degree of deviation. The innermost ring represented the GC skew, calculated as (G − C) / (G + C), where a positive value suggested a bias toward CDS transcription on the positive strand, whereas a negative value indicated preference for the negative strand.

Comparative genomic analysis of *L. garvieae* ZB15 with other *L. garvieae* strains was presented in Figure 1b. The BLAST ring comparison included *L. garvieae* strains Alexi-62, ATCC49156, DSM20684, FDAARGOS929, IBB3404, JJJN1, Lg2, 931, LG728, and MS210922A, with the outermost ring representing *L. garvieae* ZB15. This analysis revealed two distinct prophage regions: one classified as an intact phage and the other as a putative prophage. The intact prophage region exhibited a high degree of similarity to PHAGE_Lactob_ jlb1_NC024206 (46.7 kb), while the putative prophage region shared homology with PHAGE_Lactob_PLgT_NC031016 (43.4 kb) (Figure 1b). The intact prophage region encoded 58 proteins, including 34 hypothetical proteins, 17 phage-associated proteins, 3 portal proteins, 2 head proteins, 1 structural protein, and 1 domain-containing protein (Supplementary Table 1). In contrast, the putative prophage region encoded 66 proteins, comprising 51 hypothetical proteins, 5 phage-associated proteins, 3 portal proteins, 2 capsid decoration proteins, 1 ABC transporter, 1 DNA helicase, 1 tail-measuring protein, 1 minor tail protein, and 1 putative DnaC protein (Supplementary Table 2). The presence of these phage-associated genes suggested potential roles in horizontal gene transfer, genomic stability, and adaptation to environmental challenges.

**FIGURE 2:**
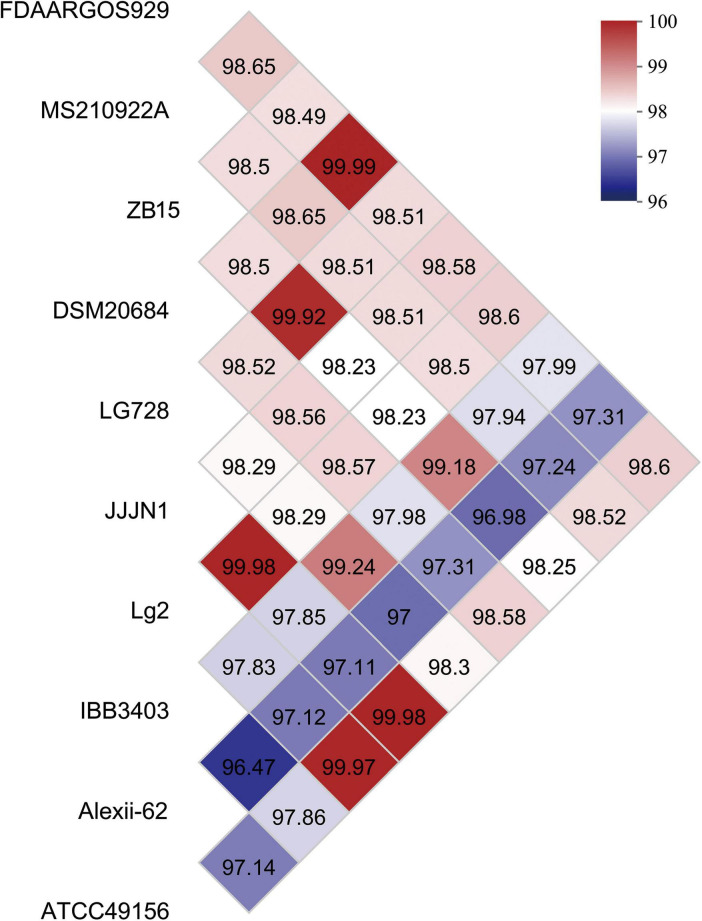
Average nucleotide identity (ANI) values of *L. garvieae* ZB15 and other compared well-known *L. garvieae* species.

To assess the genomic relatedness of *L. garvieae* ZB15 to other *L. garvieae* strains, an average nucleotide identity (ANI) analysis was conducted (Figure 2). The OrthoANI values indicated a high level of genomic similarity between *L. garvieae* ZB15 and FDAARGOS929 (98.49%), MS210922A (98.5%), DSM20684 (98.5%), LG728 (99.92%), JJJN1 (98.23%), Lg2 (98.23%), IBB3403 (98.18%), Alexi-62 (96.98%), and ATCC49156 (98.25%). Given that an ANI value above 96% was generally indicative of species-level classification, these results confirmed that *L. garvieae* ZB15 belonged to the *L. garvieae* species and shared high genetic similarity with these reference strains.

Functional annotation of *L. garvieae* ZB15 revealed 1,551 gene functions based on GO classification (Supplementary Figure 1). These functions were primarily distributed across three major categories: biological processes, cellular components, and molecular functions. The most enriched GO terms include cellular processes, metabolic processes, localization, response to stimuli, and biological regulation. Furthermore, molecular functions related to catalytic activity, binding, transporter activity, ATP-dependent activity, and transcriptional regulation were significantly represented.

**FIGURE 3:**
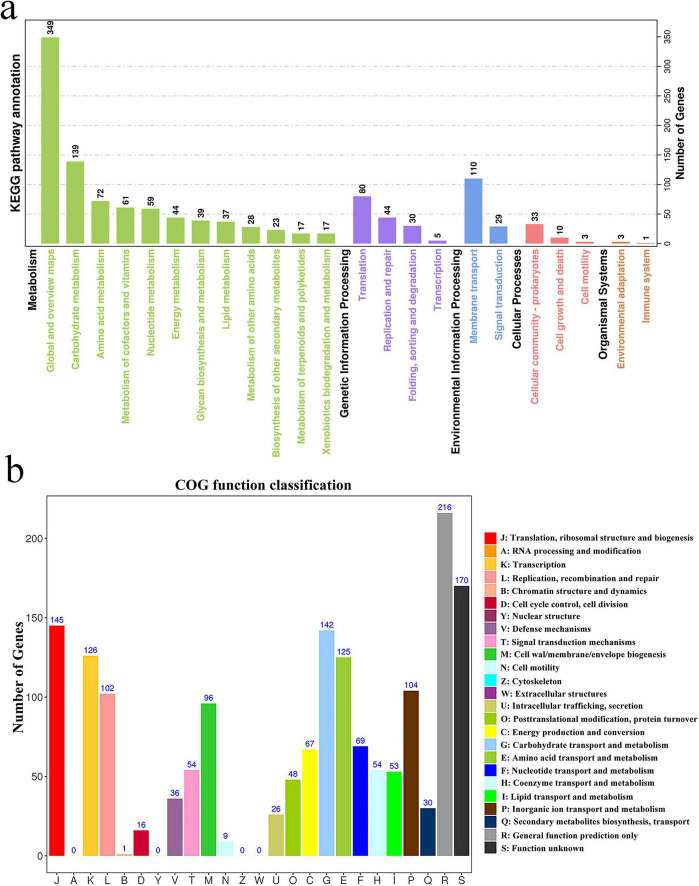
Whole-genome analysis of selected strains. **(a)** Enrichment of KEGG pathways for the *L. garvieae* ZB15 genome; **(b)** Functional classification of the *L. garvieae* ZB15 genome according to the Clusters of Orthologous Groups (COG) of proteins.

Pathway enrichment analysis using the KEGG database identified 23 significantly enriched pathways (Figure 3a). The most prominent pathways included carbohydrate metabolism, amino acid metabolism, and the metabolism of cofactors and vitamins. Additionally, genes involved in translation and membrane transport were significantly enriched, underscoring the strain’s potential metabolic versatility and environmental adaptability.

Cluster of Orthologous Groups classification further elucidated the functional profile of *L. garvieae* ZB15 (Figure 3b). The most highly represented functional categories include: translation, ribosomal structure, and biogenesis (J), RNA processing and modification (A), DNA replication, recombination, and repair (L).

These findings collectively provided insights into the genomic and functional characteristics of *L. garvieae ZB15*, suggesting its potential as a probiotic strain. The presence of phage-derived elements, diverse metabolic capabilities, and genetic similarity to other *L. garvieae* strains further highlighted its ecological adaptability and possible biotechnological applications.

### 3.2 Probiotic properties

Generally, the adhesion capacity (AC) of LAB encompasses cell surface hydrophobicity (CSH), auto-aggregation and CA capabilities. Auto-aggregation and CSH are crucial factors influencing the colonization ability of LAB in the intestinal environment. *L. garvieae* ZB15 exhibited an auto-aggregation percentage of 33.6% and a CSH of 79% (Table 2), suggesting a strong ability to adhere to intestinal epithelial cells, which is an important trait for effective probiotic function.

**TABLE 2:** The Auto-aggregation capacity and cell surface hydrophobicity of *L. garvieae* ZB15.

*L. garvieae* ZB15	Percentage/%
Auto-aggregation	33.6% ± 0.33%
Cell surface hydrophobicity	79% ± 2.14%

In addition, the CA ability of *L. garvieae* ZB15 was evaluated, as it plays a key role in inhibiting pathogenic bacteria by preventing their adhesion and biofilm formation. The strain exhibited a CA rate of 62.96% with *Escherichia coli* ATCC25922, 14.44% with *Escherichia coli* K88, 35.37% with *Staphylococcus aureus* CMCC26002, and 32.43% with *Listeria monocytogenes* CICC21635 (Table 3). These results indicate that *L. garvieae* ZB15 has a substantial ability to co-aggregate with multiple pathogens, reinforcing its potential as a probiotic strain with antibacterial properties.

**TABLE 3:** The Co-aggregation capacity of *L. garvieae* ZB15.

Pathogenic bacterium	Co-aggregation capacity
*Escherichia coli* ATCC25922	62.96% ± 1.33%
*Escherichia coli* K88	14.44% ± 0.88%
*Staphylococcus aureus* CMCC26002	35.37% ± 0.98%
*Listeria monocytogenes* CICC21635	32.43% ± 1.27%

### 3.3 Antibiotic resistance

The antibiotic susceptibility profile of *L. garvieae* ZB15 was determined using the disc diffusion method (Supplementary Table 3). The strain displayed resistance to kanamycin (30 μg), ciprofloxacin (5 μg), norfloxacin (10 μg), enrofloxacin (10 μg), vancomycin (30 μg), and sulfafurazole (300 μg). However, it was susceptible to penicillin G (10 U), ampicillin (10 μg), and amoxicillin (20 μg). Intermediate susceptibility was observed for cefotaxime sodium (30 μg), gentamicin (10 μg), erythromycin (15 μg), tetracycline (30 μg), and minocycline (30 μg).

To elucidate the genetic basis of these resistance phenotypes, whole-genome sequencing data were analyzed using the Comprehensive Antibiotic Resistance Database (CARD). No known antibiotic resistance genes were identified, suggesting that the observed resistance was likely mediated by intrinsic mechanisms rather than horizontally acquired resistance determinants. These findings indicated that *L. garvieae* ZB15 did not harbor mobile genetic elements associated with antibiotic resistance, supporting its potential safety as a probiotic candidate.

### 3.4 Carbohydrate fermentation patterns

Genomic analysis revealed that *L. garvieae* ZB15 encoded a total of 294 carbohydrate-active enzymes (CAZymes), classified into 117 glycosyltransferases, 123 glycoside hydrolases, 39 carbohydrate-binding modules, 12 carbohydrate esterases, 2 auxiliary activity enzymes, and 1 polysaccharide lyase (Supplementary Figure 2a). This extensive repertoire of CAZymes suggested a broad capacity for carbohydrate metabolism and structural modification.

Carbohydrate utilization was experimentally evaluated using the API 50 CHL assay, demonstrating that *L. garvieae* ZB15 could metabolize 17 out of 49 tested carbohydrates (Table 4 and Supplementary Figure 2b). The strain efficiently utilized D-ribose, D-galactose, D-glucose, D-fructose, D-mannose, D-mannitol, N-acetylglucosamine, amygdalin, arbutin, esculin ferric citrate, salicin, D-cellobiose, D-maltose, D-trehalose, amidon, gentiobiose, D-tagatose, and gluconate. These results highlighted the strain’s metabolic versatility and its potential adaptability to diverse nutritional environments.

**TABLE 4:** Statistics on carbohydrate metabolism ability of *L. garvieae* ZB15.

Number	Sugars	Utilization	Number	Sugars	Utilization
0	Control	**−**	25	Esculin ferric citrate	**+**
1	Glycerol	**−**	26	Salicin	**+**
2	Erythritol	**−**	27	D-Cellobiose	**+**
3	D-Arabinose	**−**	28	D-Maltose	**+**
4	L-Arabinose	**−**	29	D-Lactose	**−**
5	D-Ribose	**+**	30	D-Melibiose	**−**
6	D-Xylose	**−**	31	D-Sucrose	**−**
7	L-Xylose	**−**	32	D-Trehalose	**+**
8	Adonitol	**−**	33	Inulin	**−**
9	Methyl-β-D-xylopyranoside	**−**	34	D-Melezitose	**−**
10	D-Galactose	**+**	35	D-Raffinose	**−**
11	D-Glucose	**+**	36	Amidon	**+**
12	D-Fructose	**+**	37	Glycogen	**−**
13	D-Mannose	**+**	38	Xylitol	**−**
14	L-Sorbose	**−**	39	Gentiobiose	**+**
15	L-Rhamnose	**−**	40	D-Turanose	**−**
16	Dulcitol	**−**	41	D-Lyxose	**−**
17	Inositol	**−**	42	D-Tagatose	**+**
18	D-Mannitol	**+**	43	D-Fucose	**−**
19	D-Sorbitol	**−**	44	L-Fucose	**−**
20	Methyl-α-D-mannopyranoside	**−**	45	D-Arabitol	**−**
21	Methyl-α-D-glucopyranoside	**−**	46	L-Arabitol	**−**
22	N-Acetylglucosamine	**+**	47	Gluconate	**+**
23	Amygdalin	**+**	48	2-Keto-gluconate	**−**
24	Arbutin	**+**	49	5-Keto-gluconate	**−**

“+” means that the carbon sources can be fermented by *L. garvieae* ZB15, while “**−**” means that the carbon sources cannot be used.

### 3.5 Evaluation of antifungal activity

#### 3.5.1 Effect of cell-free supernatant (CFS) concentration on fungal growth

**FIGURE 4:**
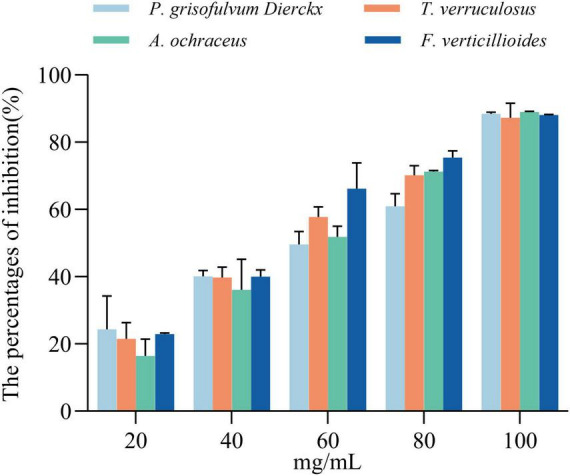
Inhibition rates of cell-free supernatant (CFS) at different concentrations against four fungal strains at 48 h. Statistical significance was determined by compared to the untreated controls, and “ns” indicates no significant difference.

The antifungal activity of *L. garvieae* ZB15 CFS exhibited a concentration-dependent effect (Figure 4 and Supplementary Figure 3). At 20 mg/mL, the inhibition rate ranged from 16.8 to 24.3%, increasing to 36.1%–40% at 40 mg/mL. A concentration of 60 mg/mL resulted in inhibition rates between 50.1 and 66.2%, while 80 mg/mL further enhanced inhibition to 60.9%–75.41%. At the highest concentration of 100 mg/mL, fungal inhibition exceeded 85%, indicating potent antifungal activity. Based on these findings, subsequent experiments were conducted using CFS at 60 mg/mL, which consistently demonstrated approximately 50% inhibition. Furthermore, Pearson correlation analysis revealed a significant positive correlation between CFS concentration and antifungal activity.

**FIGURE 5:**
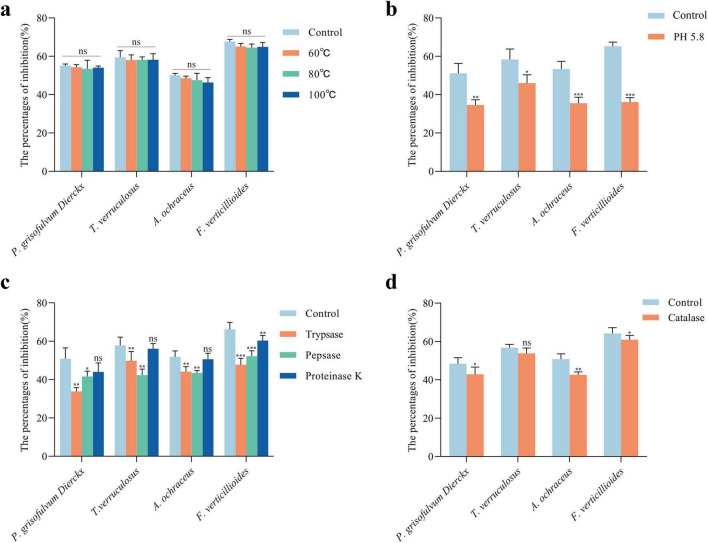
Characterization of antifungal active substances in *L. garvieae* ZB15 CFS. **(a)** Thermal stability of the CFS. **(b)** Antifungal activity of the CFS after organic acid neutralization. **(c)** Antifungal activity of the CFS following protease treatment. **(d)** Antifungal activity of the CFS after hydrogen peroxide treatment. Statistical significance was determined by compared to untreated controls. *P* < 0.05 is denoted by *, *P* < 0.01 by **, *P* < 0.001 by ***, and “ns” indicates no significant difference.

Further characterization of CFS stability showed that antifungal activity remained largely unaffected after heating at 100°C for 1 h, with only minor reductions in inhibition rates (Figure 5a). These results suggested that the antifungal compounds are heat-stable and potentially resistant to thermal degradation.

pH adjustment experiments indicated that neutralization of organic acids significantly reduced antifungal activity (Figure 5b). Increasing the CFS pH from 4.5 to 5.8 led to significant reductions in inhibition rates against *P. grisofulvum* Dierckx (33%, *P* < 0.01), *T. verruculosus* (20%, *P* < 0.05), *A. ochraceus* (33.2%, *P* < 0.001), and *F. verticillioides* (44.6%, *P* < 0.001). These findings indicated that organic acids played a key role in antifungal activity.

Protease treatments further elucidated the nature of antifungal compounds in the CFS (Figure 5c). Trypsin and pepsin treatments significantly reduced inhibition rates (*P* < 0.01), suggesting the involvement of proteinaceous components, whereas proteinase K treatment did not showed significant changes, indicating that partial proteins contributed to the antifungal effect.

Hydrogen peroxide (H_2_O_2_) degradation by catalase treatment resulted in a moderate reduction in antifungal activity (Figure 5d). The inhibition of *P. grisofulvum*, *A. ochraceus*, and *F. verticillioides* decreased after catalase treatment, with a notable 16.22% reduction in *A. ochraceus*. However, *T. verruculosum* remained unaffected, suggesting strain-specific susceptibility to H_2_O_2_.

In addition, Pearson correlation analysis revealed a significant negative correlation between treated CFS and antifungal activity. These results indicated that the antifungal activity of *L. garvieae* ZB15 CFS was mediated by a combination of organic acids, proteinaceous compounds, and hydrogen peroxide, each contributing differentially to the inhibition of fungal growth.

#### 3.5.2 Fungal morphological changes induced by CFS

**FIGURE 6:**
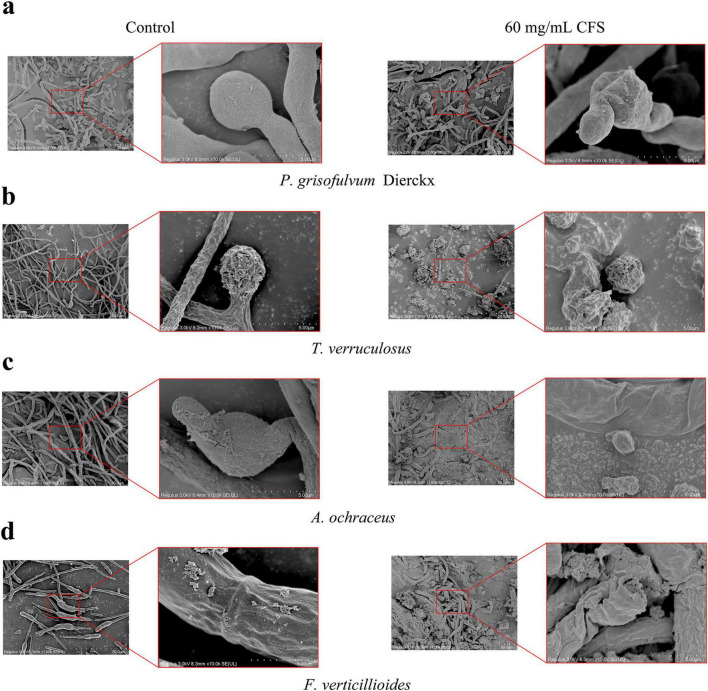
Fungal morphological changes induced by *L. garvieae* CFS. **(a)** The morphological influence of *L. garvieae* CFS on *P. grisofulvum Dierckx*. **(b)** The morphological influence of *L. garvieae* CFS on *T. verruculosus*. **(c)** The morphological influence of *L. garvieae* CFS on *A. ochraceus*. **(d)** The morphological influence of *L. garvieae* CFS on *F. verticillioides*.

The effects of *L. garvieae* ZB15 CFS (60 mg/mL) on fungal morphology were examined using scanning electron microscopy (SEM) (Figure 6). Untreated control samples exhibited normal fungal hyphal structures, characterized by smooth, elongated, and intact morphology. In contrast, CFS-treated samples showed extensive morphological disruptions, including hyphal twisting, fragmentation, and surface roughening. These alterations were evident in all tested fungal species, with varying degrees of structural damage.

Hyphal integrity loss was particularly pronounced in *P. grisofulvum* and *F. verticillioides*, where severe deformation and cell wall collapse were observed. A. ochraceus and *T. verruculosum* displayed milder but significant morphological changes, suggesting varying degrees of susceptibility among fungal species. These findings provided strong evidences that *L. garvieae* ZB15 CFS exerted antifungal effects through direct structural disruption of fungal cells, further supporting its potential as a biological control agent.

## 4 Discussion

Lactic acid bacteria have long been recognized as safe microorganisms with a history of use in fermented foods, extending their role beyond flavor enhancement to health promotion and food preservation(Wang et al., 2021). Growing research interest has focused on their ability to inhibit pathogenic microorganisms, with particular attention to the antifungal properties of LAB CFSs as potential natural preservatives.

Advancements in whole-genome sequencing have enabled comprehensive safety assessments of LAB strains. *L. garvieae* ZB15 exhibited no known virulence or antibiotic resistance genes, reinforcing its suitability as a probiotic. This finding aligns with studies on other LAB strains. For instance, *Lactobacillus paracasei* DTA93 was found to be free of virulence factors, prophages, and antibiotic resistance genes (Tarrah et al., 2020). Similarly, *Limosilactobacillus fermentum* AGA52 contained two intact prophages but lacked antibiotic resistance genes (Yetiman et al., 2024). The genome of *L. garvieae* ZB15 contained two prophages (one intact, one incomplete) but no evidence of horizontal gene transfer from different bacterial species, further confirming its genetic stability and safety for potential applications.

The ability of LAB to adhere to intestinal epithelial cells is crucial for colonization and competitive exclusion of pathogens. This property is influenced by auto-aggregation (AC), CA, and CSH (Pino et al., 2022). The 2002 *Guidelines for Evaluation of Probiotics in Food*, issued by the United Nations, state that the adhesive capacity of probiotics should encompass auto-aggregation, CA, and hydrophobicity. A high auto-aggregation capacity in LAB enables strains to attain a high density within the intestinal tract, which enhances their adhesion to the gut mucosa (Espeche et al., 2012). In this study, *L. garvieae* ZB15 exhibited an AC rate of 33.6%, comparable to previously reported values for LAB strains such as *Lactococcus lactis* and *Enterococcus species*, which ranged from 27.86 to 38.84% (Nasrollahzadeh et al., 2022).

Co-aggregation is another key probiotic trait that facilitates the inhibition of pathogen adhesion. This process facilitates the CA of pathogens with probiotic cells. Subsequently, the aggregated complexes are eliminated from the body through the gastrointestinal tract, thereby mitigating the hazards associated with pathogenic contamination (Espeche et al., 2012). *Lactococcus garvieae* ZB15 demonstrated CA with *E. coli* ATCC25922 (62.96%), E. coli K88 (14.44%), *S. aureus* CMCC26002 (35.37%), and *L. monocytogenes* CICC21635 (32.43%). These values fall within the reported range of LAB-pathogen CA rates (9.03%–96.78%) (Reuben et al., 2020), supporting the potential of *L. garvieae* ZB15 to competitively exclude pathogenic bacteria.

Cell-surface hydrophobicity represents a key physicochemical property of microorganisms. High cell-surface hydrophobicity facilitates non-specific adhesion to biotic or abiotic surfaces. Consequently, LAB exhibiting strong hydrophobicity demonstrate enhanced colonization potential within the intestinal tract (Vadillo-Rodríguez et al., 2005). CSH, a critical determinant of bacterial adhesion, was considered high when exceeding 50% (Steinberg et al., 2022). Compared to *Latilactobacillus curvatus* (13.65%) and *Weissella cibaria* (17.32%) (Zhang et al., 2022), *L. garvieae* ZB15 exhibited remarkably high hydrophobicity (79%), further underscoring its strong adhesive potential and probiotic viability.

Previous research has demonstrated that LAB-derived CFS inhibits both fungal growth and mycotoxin production. Organic acids (e.g., lactic acid, acetic acid) within CFS diffuse across the cell membrane in their hydrophobic, undissociated forms. Subsequent intracellular dissociation releases H^+^ ions, acidifying the cytosol and disrupting the proton gradient, thereby inhibiting fungal growth. Compounds such as propionic acid further reduce fungal proliferation by inhibiting amino acid synthesis or upregulating the phiA protein (Moradi et al., 2020). Beyond organic acids, antifungal peptides represent another key class of LAB metabolites. Studies indicate that antifungal peptides induce fungal cell death by binding to chitin, causing cell wall rupture, or damaging the plasma membrane, leading to leakage of intracellular constituents (Chen et al., 2023). In our study, the CFS of *L. garvieae* ZB15 exhibited concentration-dependent antifungal activity. A dose of 60 mg/mL inhibited over 50% fungal growth, while 100 mg/mL achieved suppression exceeding 80%. These findings align with results observed for *Lactiplantibacillus* MYS44, which significantly inhibited *Fusarium parasiticum* growth and aflatoxin production (Ström et al., 2002). Consistently, Gerez et al. (2010) assessed the antifungal activity of various LAB cultivated in wheat flour hydrolysate (WFH), noting inhibition of mycelial growth in *Aspergillus niger* CH 101 and *Fusarium graminearum* CH 103 by CFS from *L. plantarum* CRL 778 and *L. brevis* CRL 796.

Thermal stability is an essential characteristic of effective antifungal agents for food preservation. The antifungal activity of *L. garvieae* ZB15 CFS remained high even after heating at 100°C for 1 h, with inhibition rates ranging from 46 to 67%. This aligns with reports that *Lactococcus gasseri* and *Limosilactobacillus fermentum* CFS retained antimicrobial activity after exposure to 100°C (Scillato et al., 2021), highlighting the potential of *L. garvieae* ZB15 CFS for heat-processed food applications.

The antifungal activity of LAB CFS was attributed to multiple biologically active ingredients, including organic acids, proteins, and hydrogen peroxide (De Vuyst and Leroy, 2007). In this study, protease treatments (trypsin, pepsin, and proteinase K) significantly reduced the antifungal activity of *L. garvieae* ZB15 CFS, indicating that proteinaceous components play a key role. However, antifungal activity was not completely abolished, suggesting the presence of additional active compounds.

Acid neutralization significantly reduced inhibition rates against all tested fungal strains, confirming that organic acids mainly contributed to the antifungal effect. This was consistent with findings on *plantarum* QS494 and *Lactiplantibacillus pentosus* QS530, where acid removal diminished antifungal activity (de Lira et al., 2023). Research indicates that most LAB produce hydrogen peroxide during fermentation. However, these bacteria inherently lack the ability to produce catalase. Consequently, hydrogen peroxide accumulates in the fermented product. This compound inhibits fungal growth by oxidizing both the cytoplasmic membrane and proteins within fungal cells (Sadiq et al., 2019). Furthermore, catalase treatment reduced inhibition, indicating a role for hydrogen peroxide, similar to previous observations.

Scanning electron microscopy (SEM) revealed that *L. garvieae* ZB15 CFS caused substantial structural alterations in fungal hyphae, including fragmentation, surface roughening, and deformation. These findings were consistent with previous reports showing that LAB CFS induced severe morphological damage in fungal pathogens. *Lactiplantibacillus* MYS6, for example, was shown to cause hyphal twisting and breakage in *Fusarium proliferatum* (Deepthi et al., 2016). Similarly, *Lactiplantibacillus* YML007 disrupted fungal cell walls, leading to reduced hyphal integrity (Ahmad Rather et al., 2013). However, the precise biochemical mechanisms underlying these morphological changes remained unclear and warrant further investigations.

## 5 Conclusion

**FIGURE 7:**
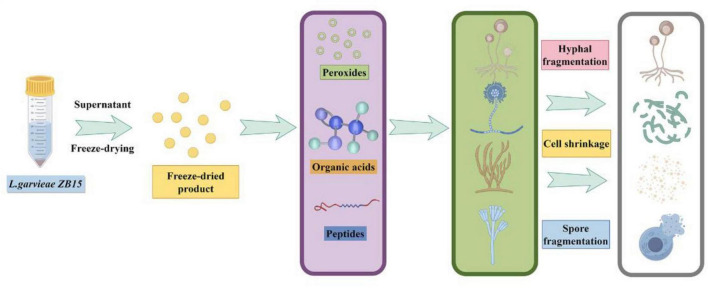
The antifungal effect of a novel *L. garvieae* derived from bacon.

This study demonstrated that *L. garvieae* ZB15 possessed probiotic characteristics, including strong adhesion properties and pathogen exclusion capabilities. Its CFS exhibited significant antifungal activity, mediated by organic acids, proteins, and hydrogen peroxide (Figure 7). The strain’s genetic safety, lack of antibiotic resistance genes, and ability to produce heat-stable antifungal compounds highlighted its potential as a natural food preservative. Future studies would focus on characterizing the specific biologically active ingredients responsible for antifungal activity and elucidating their molecular mechanisms.

## Data Availability

The original contributions presented in this study are included in this article/Supplementary material, further inquiries can be directed to the corresponding authors.
