# Supplementary material for: Assessment of genomic and antifungal properties of Lactococcus garvieae ZB15 isolated from Zhenba bacon
Source: Front Microbiol. 2025 Jun 26;16:1610971. doi: 10.3389/fmicb.2025.1610971 (PMC12241003; doi:10.3389/fmicb.2025.1610971)
Supplement: Supplementary file 1 [file Table_1.docx]

**Supplementary information**

**Figure S1 Analysis of carbohydrate metabolism capability of *L. garvieae* ZB15**

**
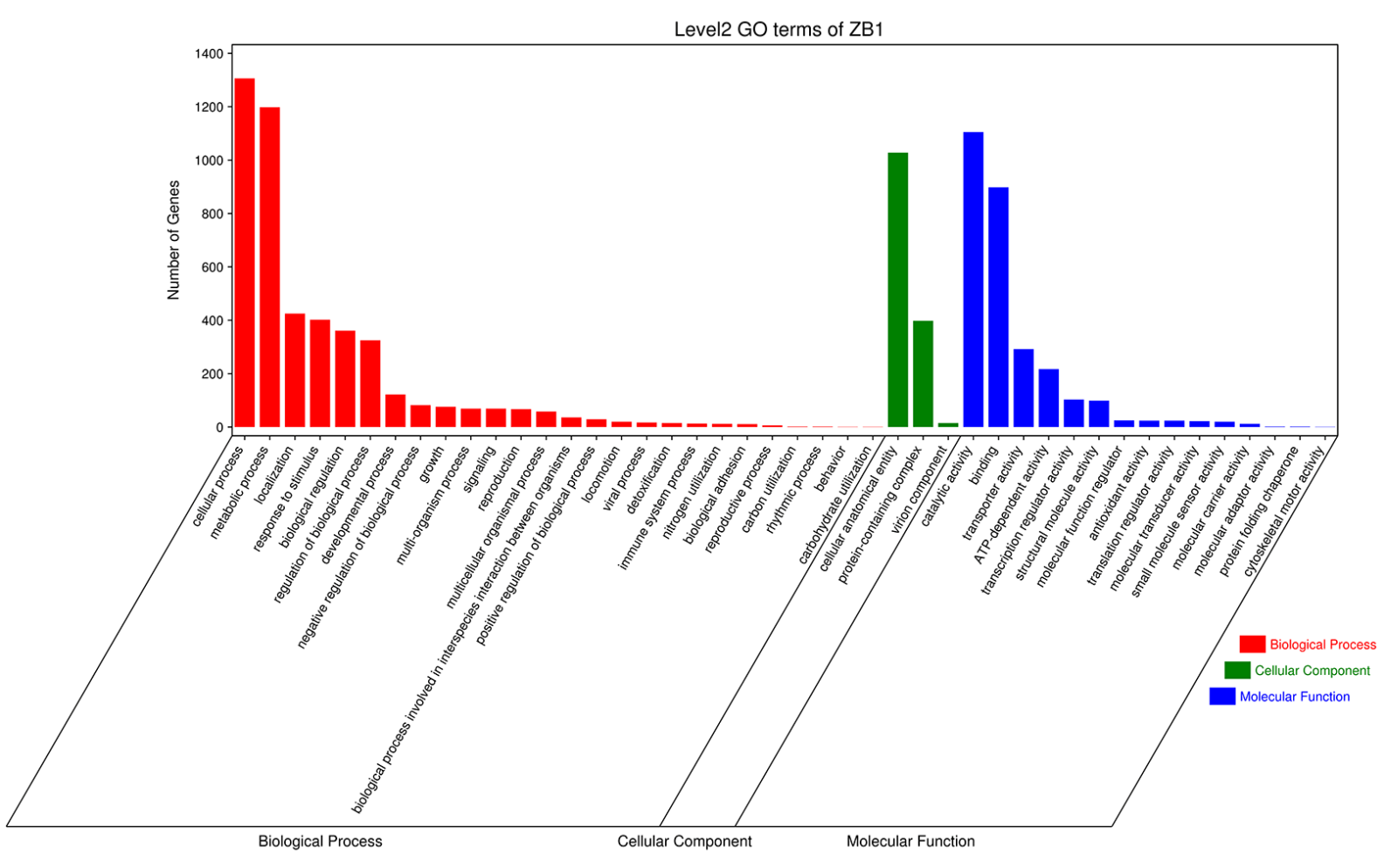
**

**Figure S1. Gene Ontology (GO) analysis of the *L.garvieae* ZB15 genome**

**
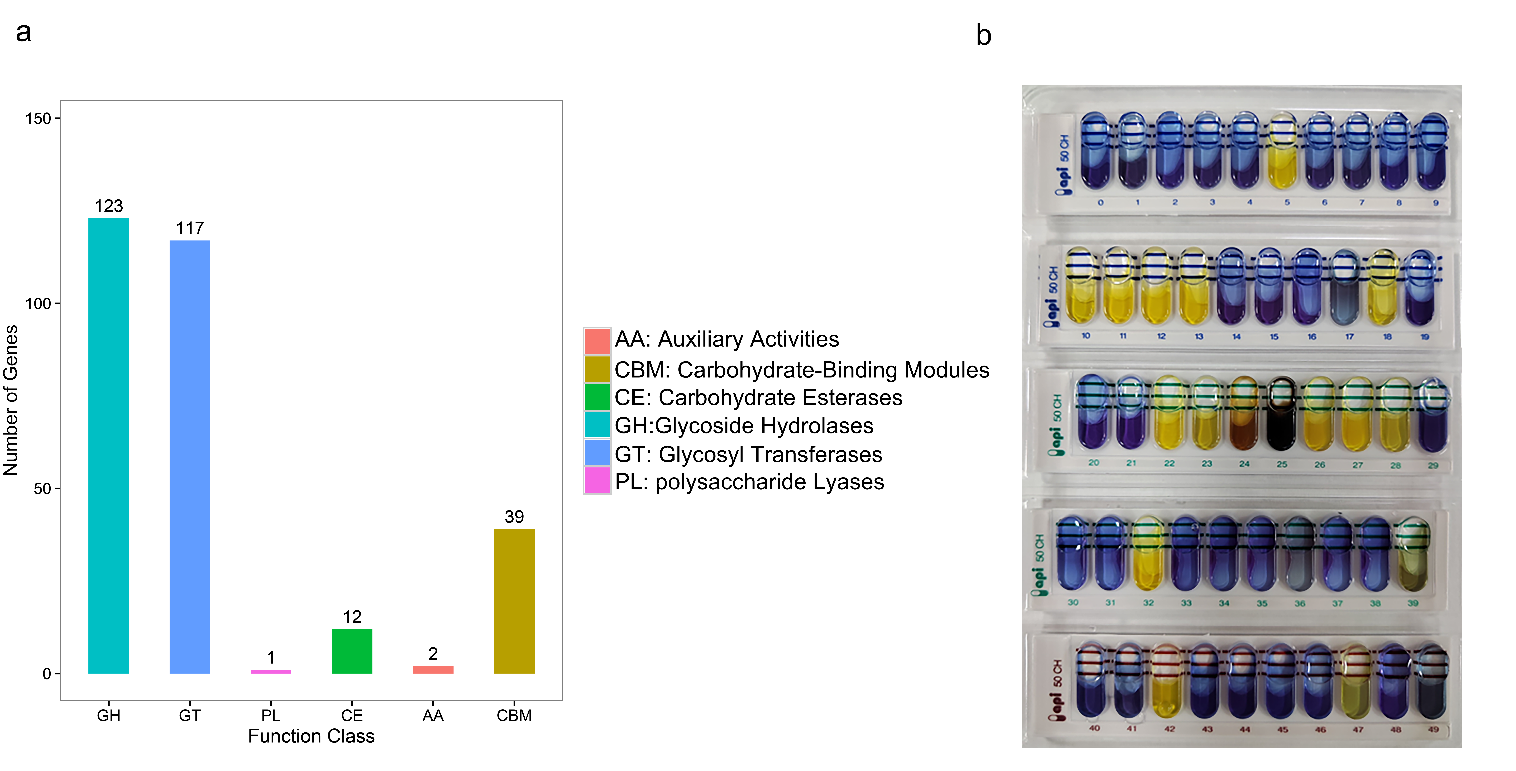
**

**Figure S2 Analysis of carbohydrate metabolism capability of *L. garvieae* ZB15**

**Figure S2. Analysis of carbohydrate metabolism capability of *L. garvieae* ZB15. a. *L. garvieae* ZB15 Carbohydrate-Active Enzymes (CAZymes) database annotation statistics chart. b. Detection Results of API 50 CHL Test Kit**

**In Figure S2b, blue indicates the carbon sources that *L. garvieae* ZB15 cannot ferment, while yellow indicates the carbon sources that can be utilized. The number 25 is the positive control with the addition of esculetin, which turns black upon acidification.**

**
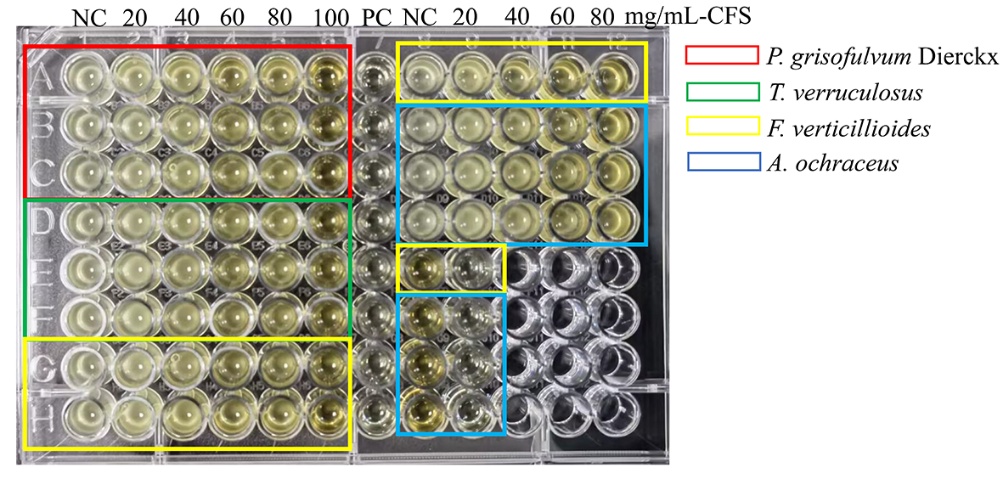
**

**Figure S3. Results of Lactic Acid Bacteria Metabolites in Inhibiting Fungi**

**NC:** **Control without CFS; 20, 40, 60, 80, 100: Experimental group with 20, 40, 60, 80, 100 mg/mL CFS added; PC: Positive control with 0.1 mg/mL fluconazole added**

**Table S1. The intact and first prophage region elements of *L. garvieae* ZB15 (PHAGE_Lactob_jlb1_NC024206).**

| **#** | **Locus** | **ORF Start** | **ORF Stop** | **Homolog/Ortholog Species** | **Homolog/Ortholog Protein** | **E-Value** |
| --- | --- | --- | --- | --- | --- | --- |
| 1 | ­­PP_00855 | 875792 | 877063 | Phage-like protein | PP_00855, gp245, phage(gi593777701), PHAGE_Bacill_G_NC_023719 | 1.69e-37 |
| 2 | PP_00856 | 877060 | 877854 | Hypothetical protein | PP_00856, hypothetical | 0.0 |
| 3 | PP_00857 | 877854 | 878654 | Hypothetical protein | PP_00857, hypothetical | 0.0 |
| 4 | PP_00858 | 878670 | 879737 | Hypothetical protein | PP_00858, hypothetical | 0.0 |
| 5 | PP_00859 | 879800 | 880936 | Phage-like protein | PP_00859, integrase; phage(gi13095806), PHAGE_Lactoc_bIL309_NC_002668 | 1.11e-119 |
| 6 | PP_00860 | 881062 | 881646 | Hypothetical protein | PP_00860, hypothetical protein; phage(gi985757753), PHAGE_Lactob_LfeSau_NC_029068 | 9.19e-25 |
| 7 | PP_00861 | 881723 | 882262 | Hypothetical protein | PP_00861, hypothetical protein; phage(gi21716074), PHAGE_Lactoc_ul36_NC_004066 | 4.60e-08 |
| 8 | PP_00862 | 882773 | 883117 | Hypothetical protein | PP_00862, hypothetical protein; phage(gi100063), PHAGE_Lactoc_PLgT_1_NC_031016 | 6.52e-42 |
| 9 | PP_00863 | 883414 | 883632 | Hypothetical protein | PP_00863, hypothetical | 0.0 |
| 10 | PP_00864 | 883646 | 883972 | Hypothetical protein | PP_00864, hypothetical protein; phage(gi21716130), PHAGE_Lactoc_ul36_NC_004066 | 1.03e-18 |
| 11 | PP_00865 | 883997 | 884719 | Structural protein | PP_00865, major structural protein; phage(gi13487806), PHAGE_Lactoc_Tuc2009_NC_002703 | 2.33e-129 |
| 12 | PP_00866 | 884741 | 884887 | Hypothetical protein | PP_00866, hypothetical | 0.0 |
| 13 | PP_00867 | 884884 | 885051 | Hypothetical protein | PP_00867, hypothetical | 0.0 |
| 14 | PP_00868 | 885498 | 885731 | Hypothetical protein | PP_00868, hypothetical | 0.0 |
| 15 | PP_00869 | 885724 | 886530 | Hypothetical protein | PP_00869, hypothetical protein; phage(gi30089870), PHAGE_Lactoc_lato_NC_004746 | 9.76e-148 |
| 16 | PP_00870 | 886524 | 887432 | Hypothetical protein | PP_00870, hypothetical protein; phage(gi30089871), PHAGE_Lactoc_lato_NC_004746 | 3.19e-149 |
| 17 | PP_00871 | 887665 | 887991 | Tail fiber protein | PP_00871, tail fiber protein; phage(gi100052), PHAGE_Lactoc_PLgT_1_NC_031016 | 1.50e-73 |
| 18 | PP_00872 | 887991 | 888797 | Hypothetical protein | PP_00872, hypothetical protein; phage(gi100051), PHAGE_Lactoc_PLgT_1_NC_031016 | 6.21e-70 |
| 19 | PP_00873 | 889079 | 889228 | Recombination protein | PP_00873, recombination protein; phage(gi100049), PHAGE_Lactoc_PLgT_1_NC_031016 | 2.64e-15 |
| 20 | PP_00874 | 889252 | 889500 | Hypothetical protein | PP_00874, hypothetical protein; phage(gi100017), PHAGE_Lactoc_P1045_NC_049812 | 1.94e-26 |
| 21 | PP_00875 | 889735 | 890016 | Hypothetical protein | PP_00875, hypothetical | 0.0 |
| 22 | PP_00876 | 890013 | 890444 | Hypothetical protein | PP_00875, hypothetical | 0.0 |
| 23 | PP_00877 | 890441 | 890746 | Phage-like protein | PP_00877, Orf28; phage(gi13095771), PHAGE_Lactoc_bIL286_NC_002667 | 2.23e-12 |
| 24 | PP_00878 | 890761 | 890952 | Hypothetical protein | PP_00878, hypothetical protein; phage(gi115315574), PHAGE_Lactoc_712_NC_00837092 | 7.41e-08 |
| 25 | PP_00879 | 890949 | 891185 | Hypothetical protein | PP_00879, hypothetical protein; phage(gi100039), PHAGE_Lactoc_PLgT_1_NC_031016 | 8.59e-13 |
| 26 | PP_00880 | 891188 | 891188 | Hypothetical protein | PP_00880, hypothetical protein; phage(gi23455740), PHAGE_Lactoc_r1t_NC_004302 | 4.76e-13 |
| 27 | PP_00881 | 891534 | 891905 | Hypothetical protein | PP_00881, hypothetical | 0.0 |
| 28 | PP_00882 | 892102 | 892518 | Domain-containing protein | PP_00882, DUF3850 domain-containing protein; phage(gi658607865), PHAGE_Lister_LP_101_NC_024387 | 5.20e-11 |
| 29 | PP_00883 | 892773 | 893393 | Phage-like protein | PP_00883, Orf36; phage(gi13095841), PHAGE_Lactoc_bIL309_NC_002668 | 1.27e-58 |
| 30 | PP_00884 | 893993 | 894442 | Hypothetical protein | PP_00884, hypothetical protein; phage(gi100021), PHAGE_Strept_phiARI0468_2_NC_031923 | 7.13e-29 |
| 31 | PP_00885 | 894432 | 894776 | Phage-like protein | PP_00885, large terminase; phage(gi208429857), PHAGE_Clostr_phiCP39_O_NC_011318 | 7.11e-22 |
| 32 | PP_00886 | 894867 | 895697 | Phage-like protein | PP_00886, putative terminase large subunit; phage(gi824479755), PHAGE_Lactoc_1358_NC_027120 | 4.79e-76 |
| 33 | PP_00887 | 895701 | 897128 | Portal protein | PP_00887, putative portal protein; phage(gi971848915), PHAGE_Lactob_jlb1_NC_024206 | 4.83e-130 |
| 34 | PP_00888 | 897349 | 898878 | Head protein | PP_00888, putative minor head protein; phage(gi971848916), PHAGE_Lactob_jlb1_NC_024206 | 1.18e-61 |
| 35 | PP_00889 | 899770 | 899113 | Phage-like protein | PP_00889, ORF34; phage(gi13786565), PHAGE_Lactoc_TP901_1_NC_002747 | 2.60e-32 |
| 36 | PP_00890 | 899341 | 899520 | Hypothetical protein | PP_00890, hypothetical | 0.0 |
| 37 | PP_00891 | 899513 | 899770 | Portal protein | PP_00891, portal protein; phage(gi100069), PHAGE_Lactob_Lenus_NC_047897 | 2.17e-21 |
| 38 | PP_00892 | 899781 | 899957 | Hypothetical protein | PP_00892, hypothetical | 0.0 |
| 39 | PP_00893 | 900158 | 900748 | Phage-like protein | PP_00893, putative minor capsid protein; phage(gi971848919), PHAGE_Lactob_jlb1_NC_024206 | 2.15e-33 |
| 40 | PP_00894 | 900748 | 901737 | Head protein | PP_00894, major head protein; phage(gi39653721), PHAGE_Strept_EJ_1_NC_005294 | 5.88e-156 |
| 41 | PP_00895 | 901874 | 902206 | Phage-like protein | PP_00895, putative major capsid protein; phage(gi971848920), PHAGE_Lactob_jlb1_NC_024206 | 1.16e-05 |
| 42 | PP_00896 | 902203 | 902454 | Hypothetical protein | PP_00896, hypothetical | 0.0 |
| 43 | PP_00897 | 902458 | 902862 | Hypothetical protein | PP_00897, hypothetical protein; phage(gi971848921), PHAGE_Lactob_jlb1_NC_024206 | 1.16e-13 |
| 44 | PP_00898 | 903217 | 903732 | Hypothetical protein | PP_00898, hypothetical protein; phage(gi971848923), PHAGE_Lactob_jlb1_NC_024206 | 2.68e-23 |
| 45 | PP_00899 | 903734 | 904165 | Hypothetical protein | PP_00899, hypothetical protein; phage(gi971848924) PHAGE_Lactob_jlb1_NC_024206 | 7.58e-11 |
| 46 | PP_00900 | 904158 | 904343 | Hypothetical protein | PP_00900, hypothetical | 0.0 |
| 47 | PP_00901 | 904343 | 905785 | Hypothetical protein | PP_00901, hypothetical protein; phage(gi971848926), PHAGE_Lactob_jlb1_NC_024206 | 1.81e-96 |
| 48 | PP_00902 | 905796 | 906254 | Phage-like protein | PP_00902, putative sheath tail protein; phage(gi971848927), PHAGE_Lactob_jlb1_NC_024206 | 2.14e-22 |
| 49 | PP_00903 | 906280 | 906687 | Phage-like protein | PP_00903, putative core tail protein; phage(gi971848928), PHAGE_Lactob_jlb1_NC_024206 | 2.06e-24 |
| 50 | PP_00904 | 906732 | 906872 | Hypothetical protein | PP_00904, hypothetical protein; phage(gi39653729), PHAGE_Strept_EJ_1_NC_005294 | 4.47e-05 |
| 51 | PP_00905 | 906880 | 910974 | Phage-like protein | PP_00905, putative minor tail protein; phage(gi971848929), PHAGE_Lactob_jlb1_NC_024206 | 1.40e-92 |
| 52 | PP_00906 | 910985 | 911665 | Hypothetical protein | PP_00906, hypothetical protein; phage(gi971848930), PHAGE_Lactob_jlb1_NC_024206 | 5.85e-51 |
| 53 | PP_00907 | 911665 | 912663 | Hypothetical protein | PP_00907, hypothetical protein; phage(gi39653732), PHAGE_Strept_EJ_1_NC_005294 | 4.98e-36 |
| 54 | PP_00908 | 912957 | 913373 | Hypothetical protein | PP_00908, hypothetical protein; phage(gi971848933), PHAGE_Lactob_jlb1_NC_024206 | 2.24e-32 |
| 55 | PP_00909 | 913370 | 914503 | Phage-like protein | PP_00909, putative baseplate protein; phage(gi971848934), PHAGE_Lactob_jlb1_NC_024206 | 2.53e-115 |
| 56 | PP_00910 | 914493 | 915044 | Hypothetical protein | PP_00910, hypothetical protein; phage(gi971848935), PHAGE_Lactob_jlb1_NC_024206 | 3.92e-16 |
| 57 | PP_00911 | 915047 | 917329 | Phage-like protein | PP_00911, u-spanin; phage(gi100032), PHAGE_Microb_Krampus_NC_047986 | 2.73e-24 |
| 58 | PP_00912 | 917357 | 917581 | Phage-like protein | PP_00912, putative holin; phage(gi14251149), PHAGE_Lactoc_BK5_T_NC_002796 | 1.20e-28 |
| 59 | PP_00913 | 917585 | 917818 | Phage-like protein | PP_00913, Holin; phage(gi23455767), PHAGE_Lactoc_r1t_NC_004302 | 6.08e-24 |
| 60 | PP_00914 | 917901 | 918569 | Phage-like protein | PP_00914, endolysin; phage(gi824479775), PHAGE_Lactoc_1358_NC_027120 | 2.08e-24 |
| 61 | PP_00915 | 919392 | 919592 | Portal protein | PP_00915, portal protein; phage(gi100069), PHAGE_Vibrio_1.026. O.10N.222.49.C7_NC_049430 | 7.56e-07 |
| 62 | PP_00916 | 920210 | 920332 | Hypothetical protein | PP_00916, hypothetical protein; phage(gi100004), PHAGE_Lactoc_PLgT_1_NC_031016 | 6.53e-21 |
| 63 | PP_00917 | 921939 | 922520 | Phage-like protein | PP_00917, transglycosylase; phage(gi849250827), PHAGE_Lactoc_WRP3_NC_027341 | 2.21e-38 |

**Table S2. The suspected and second prophage region elements of *L. garvieae* ZB15 (PHAGE_Lactoc_PLgT_1_NC_031016).**

| **#** | **Locus** | **ORF Start** | **ORF Stop** | **Homolog/Ortholog Species** | **Homolog/Ortholog Protein** | **E-Value** |
| --- | --- | --- | --- | --- | --- | --- |
| 1 | PP_01157 | 1160970 | 1161137 | Hypothetical protein | PP_01157, hypothetical protein; phage(gi100002), PHAGE_Lactoc_PLgT_1_NC_031016 | 2.17e-26 |
| 2 | PP_01158 | 1161402 | 1161584 | Hypothetical protein | PP_01158, hypothetical protein; phage(gi100003), PHAGE_Lactoc_PLgT_1_NC_031016 | 1.46e-34 |
| 3 | PP_01159 | 1162596 | 1163597 | Hypothetical protein | PP_01159; hypothetical protein; phage(gi100005), PHAGE_Strept_phiARI0131_1_NC_031901 | 3.83e-23 |
| 4 | PP_01160 | 1163811 | 1164524 | Hypothetical protein | PP_01160, hypothetical protein; phage(gi100007), PHAGE_Lactoc_PLgT_1_NC_031016 | 7.53e-58 |
| 5 | PP_01161 | 1164525 | 1164755 | Hypothetical protein | PP_01161, hypothetical protein; phage(gi100008), PHAGE_Lactoc_PLgT_1_NC_031016 | 4.02e-36 |
| 6 | PP_01162 | 1164759 | 1164965 | Hypothetical protein | PP_01162, hypothetical | 0.0 |
| 7 | PP_01163 | 1165118 | 1165387 | Hypothetical protein | PP_01163, hypothetical | 0.0 |
| 8 | PP_01164 | 1165406 | 1166581 | Hypothetical protein | PP_01164, hypothetical protein; phage(gi100010), PHAGE_Lactoc_PLgT_1_NC_031016 | 9.55e-100 |
| 9 | PP_01165 | 1166553 | 1166717 | Hypothetical protein | PP_01165, hypothetical protein; phage(gi100011), PHAGE_Lactoc_PLgT_1_NC_031016: | 6.52e-29 |
| 10 | PP_01166 | 1166701 | 1169256 | Phage-like protein | PP_01166, protein, hypothetical phage(gi100012), PHAGE_Lactoc_PLgT_1_NC_031016: | 0.0 |
| 11 | PP_01167 | 1169266 | 1169631 | Hypothetical protein | PP_01167, hypothetical protein; phage(gi100013), PHAGE_Lactoc_PLgT_1_NC_031016: | 3.89e-84 |
| 12 | PP_01168 | 1169645 | 1174336 | Hypothetical protein | PP_01168, hypothetical protein; phage(gi100014), PHAGE_Lactoc_PLgT_1_NC_031016 | 0.0 |
| 13 | PP_01169 | 1174353 | 1174721 | Hypothetical protein | PP_01169, hypothetical protein; phage(gi100015), PHAGE_Lactoc_PLgT_1_NC_031016 | 1.53e-82 |
| 14 | PP_01170 | 1174745 | 1175149 | Hypothetical protein | PP_01170, hypothetical protein; phage(gi100016) PHAGE_Lactoc_PLgT_1_NC_031016 | 9.84e-90 |
| 15 | PP_01171 | 1175396 | 1175809 | Hypothetical protein | PP_01171, hypothetical protein; phage(gi100017), PHAGE_Lactoc_PLgT_1_NC_031016 | 1.08e-85 |
| 16 | PP_01172; | 1175822 | 1176184 | Hypothetical protein | PP_01172, hypothetical protein; phage(gi100018), PHAGE_Lactoc_PLgT_1_NC_031016 | 1.01e-80 |
| 17 | PP_01173 | 1176184 | 1176726 | Hypothetical protein | PP_01173, hypothetical protein; phage(gi100019), PHAGE_Lactoc_PLgT_1_NC_031016: | 3.31e-128 |
| 18 | PP_01174 | 1176716 | 1177069 | Hypothetical protein | PP_01174, hypothetical protein; phage(gi100020), PHAGE_Lactoc_PLgT_1_NC_031016 | 1.41e-73 |
| 19 | PP_01175 | 1177050 | 1177376 | Hypothetical protein | PP_01175, hypothetical protein; phage(gi100021), PHAGE_Lactoc_PLgT_1_NC_031016 | 3.66e-68 |
| 20 | PP_01176 | 1177397 | 1178515 | Hypothetical protein | PP_01176, hypothetical protein; phage(gi100022) PHAGE_Lactoc_PLgT_1_NC_031016 | 0.0 |
| 21 | PP_01177 | 1178528 | 1179184 | Hypothetical protein | PP_01177, hypothetical protein; phage(gi100023) , PHAGE_Lactoc_PLgT_1_NC_031016 | 1.20e-136 |
| 22 | PP_01178 | 1179304 | 1179480 | Hypothetical protein | PP_01178, hypothetical | 0.0 |
| 23 | PP_01179 | 1179491 | 1179748 | Hypothetical protein | PP_01179, hypothetical protein; phage(gi100024) , PHAGE_Lactoc_PLgT_1_NC_031016 | 5.04e-55 |
| 24 | PP_01180 | 1180209 | 1180553 | Hypothetical protein | PP_01180, hypothetical | 0.0 |
| 25 | PP_01181 | 1180623 | 1181717 | Hypothetical protein | PP_01181, hypothetical protein; phage(gi100026) , PHAGE_Lactoc_PLgT_1_NC_031016 | 0.0 |
| 26 | PP_01182 | 1181723 | 1182010 | Hypothetical protein | PP_01182, hypothetical protein; phage(gi100027) , PHAGE_Lactoc_PLgT_1_NC_031016 | 5.93e-65 |
| 27 | PP_01183 | 1182007 | 1182246 | Hypothetical protein | PP_01183, hypothetical | 0.0 |
| 28 | PP_01184 | 1182288 | 1182458 | Hypothetical protein | PP_01184, hypothetical | 0.0 |
| 29 | PP_01185 | 1182415 | 1183923 | Hypothetical protein | PP_01185, hypothetical protein; phage(gi100029), PHAGE_Lactoc_PLgT_1_NC_031016 | 0.0 |
| 30 | PP_01186 | 1183933 | 1185216 | Hypothetical protein | PP_01186, hypothetical protein; phage(gi100002), PHAGE_Staphy_CNPx_NC_031241 | 0.0 |
| 31 | PP_01187 | 1185209 | 1185655 | Hypothetical protein | PP_01187, hypothetical protein; phage(gi100021), PHAGE_Strept_phiARI0468_2_NC_031923 | 1.12e-28 |
| 32 | PP_01188 | 1185889 | 1186290 | Hypothetical protein | PP_01188, hypothetical protein; phage(gi100029), PHAGE_Lactoc_98201_NC_031064 | 1.93e-13 |
| 33 | PP_01189 | 1186635 | 1186931 | Hypothetical protein | PP_01189, hypothetical | 0.0 |
| 34 | PP_01190 | 1186989 | 1187168 | Hypothetical protein | PP_01190; hypothetical protein; phage(gi849250903), PHAGE_Lactoc_WRP3_NC_027341 | 3.01e-26 |
| 35 | PP_01191 | 1187212 | 1187358 | Hypothetical protein | PP_01191, hypothetical protein; phage(gi100021), PHAGE_Lactoc_63301_NC_031017: | 6.80e-27 |
| 36 | PP_01192 | 1187546 | 1187815 | Capsid decoration protein | PP_01192, capsid decoration protein; phage(gi100066), PHAGE_Arthro_Peas_NC_048096 | 4.51e-21 |
| 37 | PP_01193 | 1187884 | 1188570 | Hypothetical protein | PP_01193, hypothetical protein; phage(gi100036), PHAGE_Lactoc_PLgT_1_NC_031016: | 1.73e-168 |
| 38 | PP_01194 | 1188592 | 1188864 | Hypothetical protein | PP_01194, hypothetical protein; phage(gi100038), PHAGE_Lactoc_ASCC281_NC_017702: | 9.74e-29 |
| 39 | PP_01195 | 1188869 | 1189288 | Phage-like protein | PP_01195; dUTPase; phage(gi13095772), PHAGE_Lactoc_bIL286_NC_002667 | 1.16e-94 |
| 40 | PP_01196 | 1189292 | 1189945 | Phage-like protein | PP_01196, u-spanin; phage(gi100032), PHAGE_Lactoc_56301_NC_049405 | 9.02e-20 |
| 41 | PP_01197 | 1189938 | 1190150 | Hypothetical protein | PP_01197, hypothetical | 0.0 |
| 42 | PP_01198 | 1190134 | 1190325 | Hypothetical protein | PP_01198, hypothetical | 0.0 |
| 43 | PP_01199 | 1190312 | 1190581 | Phage-like protein | PP_01199, endolysin; phage(gi100033), PHAGE_Lactoc_56301_NC_049405 | 5.20e-42 |
| 44 | PP_01200 | 1190639 | 1190761 | Hypothetical protein | PP_01200, hypothetical protein; phage(gi100043), PHAGE_Lactoc_PLgT_1_NC_031016 | 2.55e-17 |
| 45 | PP_01201 | 1190776 | 1191120 | DNA helicase | PP_01201, DNA helicase; phage(gi100044), PHAGE_Lactoc_PLgT_1_NC_031016 | 5.57e-78 |
| 46 | PP_01202 | 1191243 | 1191482 | Primase | PP_01202, primase; phage(gi100047), PHAGE_Lactoc_PLgT_1_NC_031016 | 1.58e-51 |
| 47 | PP_01203 | 1191870 | 1192019 | Recombination protein | PP_01203, recombination protein; phage(gi100049), PHAGE_Lactoc_PLgT_1_NC_031016 | 2.73e-07 |
| 48 | PP_01204 | 1192023 | 1192187 | Hypothetical protein | PP_01204, hypothetical | 0.0 |
| 49 | PP_01205 | 1192188 | 1193093 | Putative DnaC protein | PP_01205, putative DnaC protein; phage(gi30089873), PHAGE_Lactoc_lato_NC_004746 | 2.82e-37 |
| 50 | PP_01206 | 1193093 | 1193851 | Hypothetical protein | PP_01206, hypothetical protein; phage(gi100014), PHAGE_Lactoc_P1045_NC_049812 | 1.91e-152 |
| 51 | PP_01207 | 1193851 | 1194081 | Hypothetical protein | PP_01207, hypothetical | 0.0 |
| 52 | PP_01208 | 1194179 | 1194538 | DNA helicase | PP_01208, DNA helicase; phage(gi100044), PHAGE_Lactoc_Phi4.2_NC_049362 | 3.55e-29 |
| 53 | PP_01209 | 1194541 | 1195119 | Hypothetical protein | PP_01209, hypothetical protein; phage(gi100010), PHAGE_Lactoc_62503_NC_049811 | 5.86e-59 |
| 54 | PP_01210 | 1195122 | 1195610 | Minor tail protein | PP_01210, minor tail protein; phage(gi100055), PHAGE_Lactoc_PLgT_1_NC_031016 | 5.14e-102 |
| 55 | PP_01211 | 1195953 | 1196450 | Ail length tape-measure protein | PP_01211, tail length tape-measure protein; phage(gi100057), PHAGE_Lactoc_PLgT_1_NC_031016 | 1.89e-115 |
| 56 | PP_01212 | 1196428 | 1196661 | Hypothetical protein | PP_01212, hypothetical protein; phage(gi100058), PHAGE_Lactoc_PLgT_1_NC_031016 | 1.76e-49 |
| 57 | PP_01213 | 1196672 | 1196788 | Hypothetical protein | PP_01213, hypothetical; | 0.0 |
| 58 | PP_01214 | 1196805 | 1197098 | Hypothetical protein | PP_01214, hypothetical protein; phage(gi100059), PHAGE_Lactoc_PLgT_1_NC_031016 | 2.27e-66 |
| 59 | PP_01215 | 1197111 | 1197827 | Hypothetical protein | PP_01215, hypothetical protein; phage(gi100002), PHAGE_Lactoc_62503_NC_049811 | 5.34e-161 |
| 60 | PP_01216 | 1197858 | 1198076 | MOR | PP_01216, MOR; phage(gi13786536), PHAGE_Lactoc_TP901_1_NC_002747 | 1.05e-47 |
| 61 | PP_01217 | 1198245 | 1198787 | Hypothetical protein | PP_01217, hypothetical protein; phage(gi100004), PHAGE_Lactoc_98201_NC_031064 | 4.24e-54 |
| 62 | PP_01218 | 1198784 | 1199221 | Hypothetical protein | PP_01218, hypothetical protein; phage(gi100003), PHAGE_Lactoc_98201_NC_031064 | 4.48e-77 |
| 63 | PP_01219 | 1199272 | 1199895 | Hypothetical protein | PP_01219, hypothetical protein; phage(gi100002), PHAGE_Lactoc_50101_NC_031040 | 4.83e-39 |
| 64 | PP_01220 | 1200041 | 1201123 | Capsid decoration protein | PP_01220; capsid decoration protein; phage(gi100066), PHAGE_Lactoc_PLgT_1_NC_031016 | 0.0 |
| 65 | PP_01221 | 1201677 | 1202426 | Hypothetical protein | PP_01221, hypothetical | 0.0 |
| 66 | PP_01222 | 1202423 | 1203310 | ABC transporter | PP_01222, ABC transporter; phage(gi371496158), PHAGE_Plankt_PaV_LD_NC_016564 | 8.92e-15 |

**Table S3** **Antibiotic susceptibility test of *L. garvieae* ZB15**

| **Antibiotic Group** | **Antibiotic** | **Inhibition zone**  **diameter /status** |
| --- | --- | --- |
| β-Lactams | PEN (10U) | 16.70mm（S） |
|  | AMP (10μg) | 17.56mm（S） |
|  | AMX (20μg) | 20.43mm（S） |
|  | CTX (30μg) | 18.15mm（I） |
| Aminoglycosides | Kan (30μg) | 9.43mm（R） |
|  | GEN (10μg) | 12.62mm（I） |
| Macrolides | ERY (15μg) | 18.16mm（I） |
| Tetracyclines | TCY (30μg) | 11.94mm（I） |
|  | MNO (30μg) | 14.94mm（I） |
| Polypeptide | VA (30μg) | ≤14mm（R） |
| Sulfanilamide | SOX (300μg) | ≤12mm（R） |
| 4-Quinolones | CIP (5μg) | 10.08mm（R） |
|  | NOR (10μg) | ≤12mm（R） |
|  | ENR (10μg) | ≤14mm（R） |
